# Tight junction structure, function, and assessment in the critically ill: a systematic review

**DOI:** 10.1186/s40635-018-0203-4

**Published:** 2018-09-26

**Authors:** David Vermette, Pamela Hu, Michael F Canarie, Melissa Funaro, Janis Glover, Richard W Pierce

**Affiliations:** 1 Department of Pediatrics, Yale University, 333 Cedar Street, PO Box 208064, New Haven, CT 06520 USA; 2 Cushing/Whitney Medical Library, Yale University, 333 Cedar Street, PO Box 208064, New Haven, CT 06520 USA

**Keywords:** Tight junctions, Epithelial cells, Endothelial cells, Cellular permeability, Capillary leak, Critical care

## Abstract

**Background:**

Epithelial and endothelial barrier integrity, essential for homeostasis, is maintained by cellular boarder structures known as tight junctions (TJs). In critical illness, TJs may become disrupted, resulting in barrier dysfunction manifesting as capillary leak, pulmonary edema, gut bacterial translocation, and multiple organ failure. We aim to provide a clinically focused overview of TJ structure and function and systematically review and analyze all studies assessing markers of endothelial and epithelial TJ breakdown correlated with clinical outcomes in critically ill humans.

**Methods:**

We systematically searched MEDLINE, EMBASE, and PubMed. Additional articles were identified by targeted searches. We included studies that looked at the relationship between biomarkers of endothelial or epithelial TJ structure or function and critical illness. Results were qualitatively analyzed due to sample size and heterogeneity.

**Results:**

A total of 5297 abstracts met search criteria, of which 150 articles met requirements for full text review. Of these, 30 studies met inclusion criteria. Fifteen of the 30 reports investigated proteins of endothelial tight junctions and 15 investigated epithelial TJ markers, exclusively in the gastrointestinal epithelium. No studies investigated TJ-derived proteins in primary cardiac or pulmonary pathology.

**Conclusions:**

TJ integrity is essential for homeostasis. We identified multiple studies that indicate TJs are disrupted by critical illness. These studies highlight the significance of barrier disruption across many critical disease states and correlate TJ-associated markers to clinically relevant outcomes. Further study on the role of multiple tissue-specific claudins, particularly in the setting of respiratory or cardiac failure, may lead to diagnostic and therapeutic advances.

**Systematic review registration:**

This systematic review is registered in the PROSPERO database: CRD42017074546.

**Electronic supplementary material:**

The online version of this article (10.1186/s40635-018-0203-4) contains supplementary material, which is available to authorized users.

## Background

Tight junctions (TJs) are protein complexes that form the semi-permeable connections between cells lining corporeal compartments [1]. In endothelial and epithelial cell layers, TJs are responsible for the selective barriers that permit specialized organ function [2]. Epithelial TJs regulate alveolar air-fluid balance in the lungs, the production of appropriately concentrated urine in the kidney, as well as the absorption of nutrients and containment of bacteria throughout the gastrointestinal tract [3]. TJs in endothelia maintain intravascular volume and regulate the flux of fluid and solutes between blood vessels and organ parenchyma [4]. Endothelial and epithelial barrier dysfunction in the setting of critical illness can result in malabsorption of nutrients, translocation of gut bacteria, capillary leak, interstitial edema, tissue dysoxia, and organ failure.

Unlike other cell junctions, TJs are the structures that maintain high resistance barriers. In the setting of critical illness, generally defined as life-threatening organ failure, they may become actively dismantled [5], resulting in the capillary leak phenomenon that complicates patient management [6]. Regardless of the etiology of organ failure, many critical care practitioners believe there is likely a common pathway of active junctional disassembly resulting in capillary leak, worsening organ failure, and if not addressed, death [7]. The extent to which changes in TJ morphology, or levels of TJ-associated proteins in serum or urine, correlate to these pathophysiological processes and, more significantly, clinical outcomes remain poorly understood. In this paper, we first undertake a clinically oriented overview of TJ structure and function, followed by a systematic review of the clinical investigations studying the assessment of TJ breakdown in critically ill humans.

### General structure and function of tight junctions

#### Tight junction components and distribution

In the late nineteenth century, TJs were first identified as the physical barrier to dye diffusion at the apical surface of intestinal epithelium, dubbed the “terminal bar.” By the 1950s, TJs were recognized as key components to the barriers of frog skin and other epithelial layers [8]. Since then, TJs have been established as the primary regulators of fluid and solutes flux, based on size and charge (termed permselectivity). Research in animal models has helped to define the contributions of specific TJ proteins to tissue permselectivity and their function in disease states through knockout or overexpression studies [9]. Recent work has elucidated their roles as signaling platforms that establish cell polarity, transmit signals into nuclei, and modulate gene expression [10]. TJs are also capable of transmitting signals into cell nuclei via recruitment of adapter proteins [11]. No longer viewed as simple terminal bars, TJs are now seen as complex “plaques” containing more than 40 proteins that mechanically link adjoining cells and regulate tension, permselectivity, as well as cell signaling and gene expression [12].

**Fig. 1:**
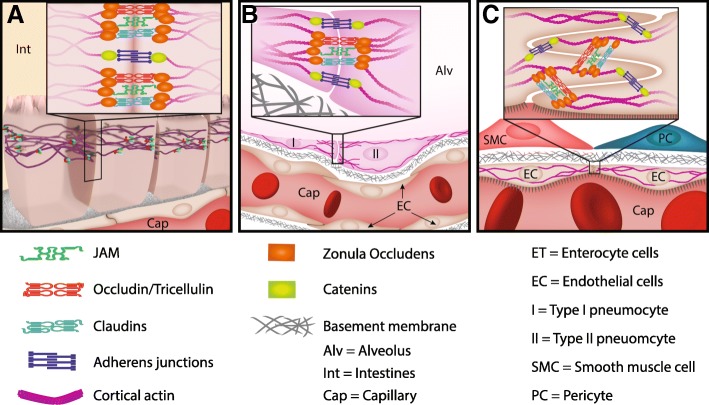
Many different cell types utilize tight junction (TJ)-dependent barriers. TJs are comprised of occludin (also called tricellulin), junctional adhesion molecules (JAMs), and claudins. Variability in the claudin component (claudin-1 through -24) dictates the permselectivity of the barrier while JAMs may also vary (JAM-A, -B, or -C), although their functional impact is less well understood. TJs are linked to the cytoskeleton through adapter proteins like zonula occludens (ZO-1, -2, and -3). **a** Columnar or cuboidal epithelia found in the upper airway, GI tract and parts of the nephron (proximal and distal tubules and collecting duct) form apical interlocking bands of tight junctions. **b** Squamous epithelia, such as in the lung, Bowman’s capsule and thin segments of the nephron, have interspaced TJs without regard to cell polarity. **c** Simple squamous endothelia of blood vessels, blood-brain barrier, and lymphatic vessels have irregularly spaced TJs that vary greatly in number based on tissue and vascular segment specific microenvironments

Although comprised of many constituents, there are three transmembrane proteins that are common to all TJs: claudins, MARVEL domain proteins, and junctional adhesion molecules (JAMs, Fig. 1) [1]. The claudin family consists of 26 members and regulates the permselectivity of specific barriers (discussed below). The MARVEL domain proteins, occludin and tricellulin, are tetra-membrane spanning proteins that regulate the recruitment of signaling complex proteins to TJs [13]. JAM-A, -B, and -C are similar to immunoglobulin-G and may play important roles in barrier formation and signaling to circulating cells (i.e., platelets via JAM-B or leukocytes via JAM-A or -C) [14].

Claudins are the main regulators of TJ permselectivity [15]. These proteins have four transmembrane passages producing two extracellular loops that may interact with claudins on the same or adjacent cells (homo-, hetero- and *cis*-, *trans*- interactions) [16]. This ability to mix and match interactions gives rise to the wide range of permselective barriers found in humans [17]. Claudin-5 promotes barrier function along with − 1, 3, 4, 8, 11, 14, and 19, while other claudin species reduce barrier function by forming ion pores like − 2 and 10. Some claudin species have ambiguous or context-specific barrier properties (− 1, 7, 12, and 15) [15].

The transmembrane components of claudin proteins link cells together while other proteins associate with the intracellular TJ plaque and facilitate intracellular signaling. Zonula occludens (ZO)-1, 2, and 3 (also known as TJ protein-1, 2, and 3) are the best studied examples [3]. ZO-1 is ubiquitously expressed in epithelial and endothelial cells and has multiple domains to facilitate cellular signaling. ZO-1 has three PDZ domains that bind claudins and JAMs, a GUK domain that binds occludin, a SH3 domain that binds the transcription factor ZONAB, and a carboxy-terminus that interacts with cytoskeletal F-actin [18]. These multiple protein interactions couple the extra- and intracellular signaling that allows the intricacy and plasticity of TJ function [10].

#### Tight junction distribution in the epithelium

Epithelial barriers are heterogeneous in their function and in the number and molecular composition of their TJs [19]. As measured by trans-epithelial electrical resistance, epithelial barrier function varies greatly among tissues, ranging from > 100,000 Ω/cm^2^ in the urinary bladder to 60 Ω/cm^2^ in the proximal jejunum [3]. This tissue-specific range of permeability is determined by the density of TJs as well as the specific claudin components [20]. Epithelial TJs exist predominately on the apical (luminal) aspect of cells and have much greater diversity of claudin expression (Fig. 1) [15]. Take for example, the nephron, where each segment has greatly different permselectivity and associated claudin expression (Table 1) [21].

**Table 1:** Predominate claudins expressed in selected tissues and tissue segments. Claudins-3 and -12 are especially concentrated in the cerebral endothelium

Tissue	Predominate claudin species
Kidney	
Glomerulus	-1, 2, 6
Proximal tubule	-2, 6, 9, 10, 17
Thin limb	-4, 7, 8, 10
Thick limb	-10, 14, 16, 19
Collecting duct	-3, 4, 7, 8, 10, 18
Lungs	
Conducting airways	-1, 3, 4, 8
Respiratory airways	-3, 4, 18
Gastrointestinal tract	
Stomach	-2, 3, 4, 10, 13, 18
Small intestine	-3, 4, 5, 18
Intestinal crypt	-2, 10, 15
Intestinal villi	-3, 4, 7, 8, 13, 23
Colon	-3, 4, 7, 8
Endothelium	-3, 5, 12

Similarly, pulmonary epithelial cells express claudin species with regional diversity (Table 1). The pseudo-stratified columnar epithelium of the conducting airway (above generation 16) requires fluid flux for mucociliary transport. The squamous epithelium of the respiratory airways requires a tightly regulated thin layer of surfactant to maintain alveolar spaces for gas exchange [22]. At the alveolar level, there is greater variability of claudin expression in TJ formed by type 2 pneumocytes compared to type 1, reflecting their more variable functions [23].

Cell junctions in the gastrointestinal (GI) tract are perhaps the most complex, with variation along the length as well as within crypt-villi axes, reflecting the variable functions of the gut (Table 1) [24]. The GI barrier is comprised of intimately related endothelial and epithelial cells which depend on numerous, tract-specific claudins to selectively allow nutrient absorption while excluding bacteria [19]. Additional claudin species are likely expressed under special circumstances such as acute disease [25]. The spatial and temporal heterogeneity of claudin expression permits the range of permeability essential to GI function and adaptability. Unfortunately, this wide range of expression may limit the utility of these proteins as tissue-specific biomarkers.

#### Tight junction distribution in the endothelium

Components of endothelial TJs are generally conserved throughout the endothelium. TJs are interspersed throughout areas of cell-cell overlap with little regard for cell polarity (Fig. 1). Regional permselectivity is established by varying the density and distribution of TJs between vascular segments [26]. The slightly permeable artery, arteriole, and large vein segments contain high numbers of regularly spaced TJs, whereas post-capillary venules typically have very few [27]. The permeability of the capillary segment is exquisitely organ specific, with highly permeable capillaries in the liver sinusoids that have fewer and more irregularly spaced TJs than less permeable capillaries such as those that line alveoli [28]. The least permeable capillaries are those that form the specialized blood-brain barrier [29].

The density and identity of claudins species dictate the permselectivity of organ-specific vascular segments. Claudin-5 is expressed by all endothelial cells. Claudins-1, 3, and 12 are also present in vascular endothelial TJs albeit at low levels. The blood-brain barrier is established by numerous TJs containing large amounts of claudins-3, 5, and 12 [29]. Occludin and zonulin (also known as prehaptoglobin-2) are expressed throughout the epithelium and endothelium while JAM-A and JAM-C are restricted to endothelium.

### Systematic review of the assessment of tight junction breakdown in critically ill humans

Despite their importance, there has been little clinical research regarding how TJs respond to critical illness and how damage to specific corporeal barriers can be monitored [4]. Better assessments of TJ dysfunction in critical illness could help guide common therapies such as volume resuscitation, ventilator management, antibiotic therapy, or diuretic regimens and aid in the development of novel therapies that specifically target TJs. To define the state of the TJ-derived protein biomarker assessment in clinical research, we performed a systematic review and qualitative analysis of all investigations that assess TJ structure and function in critically ill humans.

## Methods

We followed the Preferred Reporting Items for Systematic Reviews and Meta-Analyses. Our study protocol is registered in the PROSPERO database of systematic reviews, CRD42017074546 (Additional file 1: Table S1).

### Search strategy

The authors conducted a systematic review, searching MEDLINE (Ovid MEDLINE 1946 to August Week 2 2017), Embase (Ovid Embase 1974 to 2017 August 21), and PubMed (through August 22, 2017) without language restriction. Searches in Embase and MEDLINE were limited using the human filter. Additional articles were identified by examining other systematic reviews, reference lists, bibliographies, and pre-identified websites such as research grant databases (NIH reporter), patent searches (USPTO search), conference abstracts (Web of Science), and publicly available internet searches (Google Scholar). Search strategy and terms, including both controlled vocabulary terms and free-text terms for the concepts of critical illness and tight junctions, are listed in Additional files 2: Tables S2 and Additional file 3: Table S3.

On January 3, 2018, March 30, 2018, and June 11, 2018, updated searches were completed in MEDLINE, Embase, and PubMed. The search repeated the controlled vocabulary terms (Additional files 2: Tables S2 and Additional file 3: Table S3) and free text terms for the concept “critical illness” and then added terms for “tight junctions” and references were screened and selected as described.

### Study selection

**Fig. 2:**
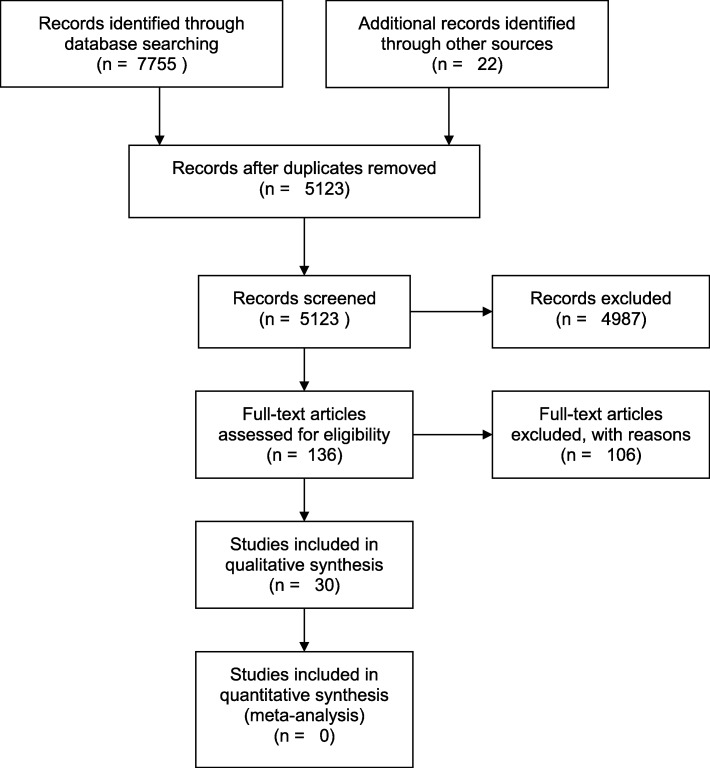
Search strategy and results of the systematic review of the literature

Two independent, blinded reviewers completed title, abstract, and full text screens of the results (Fig. 2). The senior author mediated consensus meetings to resolve discrepancies. Eligible studies include those assessing TJ structure or function in critically ill humans. Articles were screened for meeting the inclusion criteria: (1) observational case report, cohort, case control, or clinical trial studies; (2) studies involving critically ill human patients; and (3) studies assessing tight junction biomarkers or pathology (Additional files 2: Tables S2 and Additional file 3: Table S3). The determination of a patient as “critically ill” has some inherent variability, so we relied on the clinical impressions and determinations of the primary authors of each study. Studies investigating animal or human cellular models (in vitro and ex vivo) as well as those that did not specifically assess TJ-derived protein components were excluded.

Studies examining barrier disruption due to cell apoptosis were excluded as this represents a different pathophysiologic process from the active TJ remodeling and cell junction disassembly of interest here [30–32]. Studies that evaluated the protein ZO-1 at intercalated discs of cardiomyocytes were also excluded since, although they contain protein ZO-1, these structures themselves are not TJs [33, 34].

### Assessment of the evidence and data abstraction

All collected studies were reviewed using the Grades of Recommendation, Assessment, Development and Evaluation (GRADE) criteria from the Cochrane Review group [35]. A quantitative meta-analysis was not performed for several reasons. Relative estimates of effect could not be calculated for case series and studies that lacked control groups. Methodological heterogeneity, such as the type of tissue or fluid sampled, the timing of sampling relative to disease onset, and the wide range of analytical techniques employed (even between studies on the same molecule), further prohibited quantification of results. Therefore, all studies were qualitatively analyzed.

## Results

A total of 5297 abstracts met search criteria for screening of which 150 articles satisfied the requirements for full text review. Of those, 30 studies were included that investigated biomarkers of epithelial (15 studies) and endothelial (15 studies) TJ structure or function in critically ill humans.

Overall, the quality of the identified evidence for using epithelial and endothelial biomarkers to predict outcomes is low. The main limitations were study design, as no randomized controlled trials were identified and only observational trials could be included. Moreover, these studies in general had low numbers of subjects and lacked control groups; 19 of the 30 studies identified included control patients while 18 of the 30 studies included involved 25 or less subjects. Only 2 identified studies included more than 100 subjects. Although trends were identified across studies, there was discordance in results for ZO-1 and claudin-2 in tissue samples between studies, likely due to different tissue and timing of collection relative to disease onset. Interpretation of pathology specimens was predominately qualitative, performed by individual authors, and primary data, such as complete histologic collections, could not be completely reviewed. The overall lack of standardization of tissue sample size, collection time, detection methods, and presentation of complete histologic datasets further downgraded the quality of the available evidence.

### Epithelial injury

Fifteen studies looked at epithelial TJs, all focusing on TJ regulation in the GI tract during critical illness (Table 2). Of these, 13 were prospective observational and 2 were cross-sectional studies. Nine studies examined biopsy specimens without a consistent approach to the timing of biopsies relative to onset of illness or segment of bowel sampled. Despite this heterogeneity, these studies collectively demonstrate that TJs become disrupted in response to critical illness. In concordance with the histologic findings, multiple markers of TJ disruption were detected in blood or urine. However, these studies must be carefully interpreted within their individual clinical contexts as zonulin is widely expressed in most cell types while claudins-3 and -4 may be more specific for EC injury. Notably, no studies specifically evaluated TJ proteins enriched in pulmonary or renal epithelium. Seven studies investigated GI-derived TJ markers, in the settings of severe malnutrition [36], non-GI surgery [37], congenital heart surgery requiring cardiopulmonary bypass [38, 39], and general critical illness [40–42]. All together, these studies found disrupted TJs as assessed by histology or electron microscopy. Combining a variety of investigative methods, including histology or electron microscopy, these studies collectively indicate that TJs in the GI tract are disrupted in critical illness. Decreased serum levels of claudin-4 were found in malnutrition states [36]. Decreased claudin-1, but not claudin-2 or occludin, was detected after emergent colectomy [41]. Urinary and plasma claudin-3 concentrations increased after major surgery and correlated with clinical parameters [37–39]. Mechanically ventilated patients had increased levels of serum zonulin associated with decreased gastric motility [40] and, among those without overt GI pathology, had generally intact TJ ultrastructure [42].

**Table 2:** Summary of studies investigating markers of epithelial injury listed in descending chronological order

Study	Study design	Disease process	Method of TJ assessment	# of Pts (controls)	TJ proteins studied	Major findings
Amadi et al. 2017 [36]	Prospective observational study	Severe acute malnutrition	Intestinal biopsy; IHC	34 (0)	Claudin-4	Reduced claudin-4 tissue staining overall, with increased staining and more disorganization at epithelial breaks and villus tips
Bein et al. 2017 [71]	Cross-sectional study	NEC	RT-PCR; IHC	6 (6)	ZO-1, occludin, claudin-4, cingulin	Decreased expression of occludin and ZO-1 in the jejunum and ileum of NEC patients
Goswami et al. 2017 [72]	Prospective observational study	SAP	Duodenal biopsy; IHC; RT-PCR; EM	26 (10)	Claudin-2, -4	Decreased claudin-4 in villi and crypts; Increased expression of claudin-2 and disruption of TJ by EM in SAP
Greis et al. 2017 [40]	Prospective observational study	Intestinal dysfunction in mechanically ventilated patients	Serum ELISA	50 (0)	Zonulin	Increased serum zonulin (2.5 10.4 ng/mL) associated with delayed gastric emptying
Habes et al. 2017 [38]	Cross-sectional study	Intestinal injury after cardiac surgery	Urine ELISA	37 (0)	Claudin-3	Urinary claudin-3 (0 to 0.23 ng/mL) increased at onset of surgery, peaked at end of surgery and normalized over several days
Sipola et al. 2017 [41]	Prospective observational study	Emergent colectomy	Colectomy samples; IHC	38 (28)	Claudin-1, -2; occludin	Decreased claudin-1 tissue staining in the damaged colon of critically ill patients. No significant changes seen with claudin-2 or occludin expression
Tarko et al. 2017 [45]	Prospective observational study	NEC, rotavirus and gastroschisis	Serum ELISA	67 (14)	Zonulin	Increased serum zonulin (2 to 43 ng/mL) levels in patients with rotavirus and gastroschisis
Wen et al. 2017 [47]	Prospective observational study	SAP	Colonic biopsy; IF; WB	31 (8)	Claudin-2; occludin; ZO-1	Increased claudin-2 and decreased occludin and ZO-1 tissue staining associated with bacterial translocation
Liew et al. 2016 [42]	Prospective observational study	Mechanically ventilated adults	Duodenal biopsy, EM	12 (15)	Not specified	No abnormalities of TJ ultrastructure were appreciated in the duodenal samples of patients in either the critically ill group or control group
Sonika et al. 2016 [48]	Prospective observational study	SAP	Duodenal biopsy; IHC	20 (20)	Claudin-2, −4	Significantly decreased tissue staining of claudin-4 in duodenal villi and crypts but not significantly decreased at intercellular junctions. Claudin-2 had no statistically significant changes
Blackwood et al. 2015 [43]	Prospective observational study	NEC	Intestinal biopsy IF; urine WB	3 (3)	Claudin-2	Reduced claudin-2 tissue staining with an increase in urinary claudin-2
Typpo et al. 2015 [39]	Prospective observational study	Intestinal dysfunction after CPB	Serum ELISA	20 (0)	Claudin-3	Increased plasma claudin-3 (0 to 18 ng/mL) levels more than 120 h after CPB were associated with fluid overload, feeding intolerance and duration of antibiotic treatment
De Plaen et al. 2012 [46]	Prospective observational study	NEC	Colonic and small intestine biopsy; IHC	20 (20)	Claudin-2, -4; occludin; ZO-1	Increased tissue staining of claudin-2 but not of claudin-4, occludin, or ZO-1
Thuijls et al. 2010 [44]	Prospective observational study	NEC	Urine WB	14 (21)	Claudin-3	Increased urinary claudin-3 in patients who developedNEC
Derikx et al. 2008 [37]	Prospective observational study	Intestinal injury after non-GI surgery in children	Urine WB	20 (0)	Claudin-3	Increased urine claudin-3 associated with onset of surgery and rapid return to baseline levels

*IHC* immunohistochemistry, *IF* immunofluorescence, *EM* electron microscopy, *WB* Western blot, *ELISA* enzyme-linked immunosorbent assay, *NEC* necrotizing enterocolitis, *SAP* severe acute pancreatitis, *CPB* cardiopulmonary bypass, *IP* intestinal permeability, *CSF* cerebral spinal fluid, *GI* gastrointestinal, *RT*-*PCR* reverse transcription polymerase chain reaction, *ZO*-*1* zonula occludens, *NR* not reported

Five studies investigated TJs in infants with necrotizing enterocolitis (NEC). Urinary claudin-2 [43] and -3 [44] and plasma zonulin [45] are elevated in this setting and correlate temporally with episodes of NEC. Tissue levels of claudin-2 are also altered in NEC, with increased staining in the colon [46] and decreased staining in the intestine [43]. Expression of occludin and ZO-1 in the jejunum and ileum of patients with NEC was found to be decreased (31).

Lastly, three studies investigated TJ biomarkers in colonic tissue from patients with severe acute pancreatitis (SAP). These studies demonstrated decreased expression of ZO-1, occludin, and claudin-4 in either duodenal or colonic tissue [47–49]. The magnitude of decreased ZO-1 expression correlated with the degree of bacterial translocation [47, 49].

### Endothelial injury

Fifteen studies, of which 9 were prospective observational, 5 were cross sectional, and 1 was a case report, evaluated endothelial TJ proteins in the setting of neurologic injury, sepsis, and trauma (Table 3). There were three studies on ischemic stroke [50–52], and one study on each of the following: intracranial hemorrhage [53], cerebral edema [54], kernicterus [55], cerebral malaria [52], and fatal heat stroke [56], two studies on trauma [57, 58]. There were an additional five studies on sepsis [59–63]. However, claudin-5 is restricted to EC, while ZO-1, occludin, JAM-A, vinculin, and zonulin are expressed in other cells, and elevated levels may not reflect TJ breakdown. Therefore, interpretation of studies of these proteins must take into account the specific clinical context and be correlated with other cellular injury.

**Table 3:** Summary of studies investigating markers of endothelial injury listed in descending chronological order

Study	Study design	Disease process	Method of TJ assessment	# of Pts (controls)	TJ proteins studied	Major findings
Aslan et al. 2017 [59]	Cross-sectional study	Severe sepsis	Post-mortem kidney biopsy; RT-PCR	19 (12)	Claudin-5; occludin	Increased claudin-5 mRNA expression associated with sepsis and no significant difference in occludin mRNA expression
Du et al. 2017 [56]	Cross-sectional study	Fatal heat stroke	Post-mortem brain biopsy; IHC; RT-PCR	23 (23)	Claudin-5; occludin; ZO-1	No significant difference in staining of claudin-5, occludin or ZO-1 between heat stroke and control group
Halbgebauer et al. 2017 [57]	Prospective observational study	Polytrauma	Serum ELISA	30 (0)	Claudin-5	Increased serum claudin-5 (20 to 400 ng/mL) levels associated with hemorrhagic shock, serum lactate and need for transfusion
Ji et al. 2017 [50]	Randomized prospective study	Ischemic stroke	Serum ELISA	16 (14)	Claudin-5; ZO-1	No difference in claudin-5 or ZO-1 (NR) between treatment groups in the acute phase, but decreased serum claudin-5 (338 versus 408 ng/mL) in the treatment group after 10 days
Suwarto et al. 2017 [60]	Prospective observational study	Dengue fever	Serum ELISA	103 (0)	Claudin-5	Significantly increased serum claudin-5 (17.4 to 81.1 ng/mL) levels associated with dengue with severe plasma leakage as compared to dengue fever and dengue hemorrhagic fever
Zhao et al. 2016 [61]	Cross-sectional study	Sepsis	Serum ELISA	51 (0)	Claudin-5; occludin; ZO-1	Increased serum occludin (285versus 607 pg/mL) and ZO-1 (54 versus 735 pg/mL) in severe sepsis and septic shock and negatively associated with survival. Increased serum ZO-1, but not occludin, associated with multiple organ dysfunction syndrome. No significant differences found in claudin-5 levels (281 versus 263 pg/mL) between survivors and non-survivors and MODS and non-MODS groups
Denk et al. 2015 [58]	Prospective observational study	Polytrauma	Serum ELISA	8 (10)	JAM-A	Increased serum JAM-A (4 to 10 ng/mL) correlated with APACHE-II and SOFA scores
Jiao et al. 2015 [53]	Prospective observational study	Intracranial hemorrhage (ICH)	CSF and serum ELISA	22 (17)	Claudin-5; occludin; ZO-1	Increased claudin-5, occludin, and ZO-1 in the CSF (1302, 10, 934 versus 157, 0.5, 181 pg/mL respectively) but not in the serum (89, 13, 2008 versus 126, 11, 2173 pg/mL respectively) of ICH patients compared to controls. CSF TJ proteins have higher sensitivity and specificity in diagnosis of ICH than serum proteins
Wichapoon et al. 2014 [62]	Cross-sectional study	*Plasmodium falciparum* malaria	Post-mortem kidney biopsy; IHC	20 (10)	ZO-1	Decreased ZO-1 expression associated with acute kidney injury in malaria patients
Klaus et al. 2013 [63]	Prospective observational study	Sepsis	Serum ELISA	25 (38)	Zonulin	Increased serum zonulin (3.4 versus 6.6 ng/mL) levels in septic patients compared to control patients
Brito et al. 2012 [55]	Case report	Kernicterus	Post-mortem brain biopsy; IHC	1 (1)	Claudin-5	Increased claudin-5 tissue staining in the kernicteric patient
Kazmierski et al. 2012 [51]	Prospective observational study	Ischemic stroke	Serum ELISA	458 (0)	Claudin-5; occludin; ZO-1	Increased serum occludin (0 to 0.08 pg/mL) and claudin-5/ZO-1 ratio (0 to 1.47 and 0.57 to 1.48 RU/mL respectively) is associated with hemorrhagic conversion and correlates with markers of neuron injury (S100B)
Haarmann et al. 2010 [52]	Prospective observational study	Ischemic stroke and multiple sclerosis	Serum ELISA	13 (45)	JAM-A	No significant change in serum JAM-A (7 to 15 ng/mL) level over time in ischemic stroke
Brown et al. 1999 [64]	Prospective observational study	Cerebral malaria	Post-mortem brain biopsy; IHC	14 (10)	Occludin; vinculin; ZO-1	Decreased tissue staining of ZO-1, occludin, and vinculin
Castejon OJ 1980 [54]	Cross-sectional study	Cerebral edema in trauma, tumor or malformation	Brain biopsy; EM	17 (0)	Zonulin	Endothelial junctional morphology is altered in moderate and severe cerebral edema

*CSF* cerebral spinal fluid, *ELISA* enzyme-linked immunosorbent assay, *EM* electron microscopy, *IHC* immunohistochemistry, *JAM* junctional adhesion molecule, *MODS* multiple organ dysfunction syndrome, *RT*-*PCR* reverse transcription polymerase chain reaction, *ZO* zonula occludens, *NR* not reported

The largest study identified included 458 patients and found that the serum levels of claudin-5, occludin, and the claudin-5-to-ZO-1 ratio were significantly increased in patients with hemorrhagic conversion of ischemic stroke [51]. These markers demonstrated high negative predictive value in this setting as well, specifically occludin (95.5%), claudin 5 (94.5%), occludin-to-claudin-5 ratio (88.9%), ZO-1 (94.3%), and claudin-5-to-ZO1-ratio (95.9%)**.** In other studies of stroke [52], JAM-A, ZO-1, and claudin-5 were found not to correlate with blood-brain barrier breakdown in the acute phase; however, a decrease in claudin-5 levels may indicate improved barrier function by 10 days. In the largest study identified [51], increased serum occludin and elevated claudin-5 to ZO-1 ratio was associated with worse hemorrhage and neuronal injury (as measured by S100B levels). In intracranial hemorrhage, claudin-5, occludin, and ZO-1 sampled from cerebrospinal fluid may be more specific than serum markers [53].

Changes in vascular TJ architecture have been associated with cerebral edema. A study looking at electron microscopy of autopsy specimens demonstrated TJ disruption in cerebral edema due to hemorrhagic congenital malformations, brain tumors, or trauma [54]. Claudin-5 is increased in metabolic encephalopathies such as kernicterus [55]. In the setting of infection such as cerebral malaria, ZO-1, occludin, and vinculin are downregulated [64]. Lastly, in the setting of fatal heat stroke, serum claudin-5, ZO-1, and occludin levels are unchanged [56]. No studies have investigated blood-brain barrier-specific claudins in critical injury.

Systemic endothelial disruption in the setting of trauma was investigated in two studies. First, in polytrauma patients, JAM-A is significantly elevated within 4 h and correlated with clinical severity scores and outcomes [58]. Second, in patients with trauma-induced hemorrhagic shock, claudin-5 is elevated and correlated with serum lactate, indicating that endothelial damage may correlate with systemic oxygen delivery [57].

Five studies have investigated TJs in sepsis, with three investigating serum biomarkers and two focusing on renal biopsies in sepsis-associated kidney failure. Collectively, these studies demonstrate that serum levels of ZO-1, occludin, zonulin, and claudin-5 are elevated in sepsis [59, 61, 63]. ZO-1 may be more predictive of multiple organ dysfunction and mortality than claudin-5 or procalcitonin in septic patients presenting to the emergency department [61]. Likewise, in the setting of Dengue fever, claudin-5 levels correlate with the degree of clinical plasma leakage [60]. In sepsis-induced kidney injury, claudin-5 may be increased and ZO-1 decreased in endothelial cells of biopsy specimens [59]. However, no kidney-specific epithelial claudins were assessed.

Notably, no studies investigated multiple organ failure using organ-specific TJ biomarkers in critically ill humans. Furthermore, no studies investigated endothelial TJ structure or function in the context of primary cardiopulmonary disease or failure.

## Discussion

The maintenance of functional tissue barriers is essential to life and the principal functional components of these barriers are TJs. There is great complexity in TJ structure, amplified by their heterogeneity across tissues types (Fig. 1). To best assess these complex, dynamic processes, and determine their utility as potential biomarkers, we combined the limited histologic and biomarker studies available. Our review identifies growing evidence that TJs are disrupted in critical illness and markers of their breakdown do correlate to clinically significant outcomes across multiple disease states, in clinically relevant time points. However, many questions remain as the overall number of identified studies is small and they are of variable quality.

The epithelium comprises a diverse set of tissue barriers, separating air, stool, urine, and all the elements from the interstitium. This functional complexity is reflected in tissue-specific molecular composition (Table 1). Studies involving epithelial damage described acute injury to GI tract, and over half of those studies investigated tissue samples. These studies reveal that TJ proteins are downregulated in critical illness, weakening the GI barrier. However, broader interpretation of these results is limited by the heterogeneous techniques employed, variable timing of TJ assessment with respect to disease presentation and overlapping patterns of protein expression.

Results of epithelial TJ markers from urine and blood, however, are more promising and the collected studies indicate useful clinical applications. These results consistently indicate that serum or urine levels of claudins-2, 3, and 4 correlate with acute GI injury. Although these species are expressed in other tissues, in the clinical context of GI injury, they may prove to be valuable biomarkers. Urinary claudins were an early and reliable measure of GI damage and permeability across several disease processes from NEC to post-surgical GI insults, and could provide clinicians with minimally invasive assessments of gut integrity in real time. Plasma zonulin levels may also be useful in diagnosis of NEC, although they are more significantly elevated in other GI pathologies such as gastroschisis. However, as many of these proteins are also expressed in the kidney and lungs, more research is needed to refine them as tools of tissue-specific injury.

Multiple studies investigated levels of ZO-1, occludin, or zonulin, without concurrent investigation other, more specific of markers of TJs. The aforementioned proteins are expressed in many cells types, potentially confounding their interpretation. Elevation of these proteins in critical illness may reflect a combination of endothelial, epithelial, or organ parenchymal damage. Similar to study of the epithelium, these investigations were a mix of histological examination and measurement of serum protein levels. The six histologic studies identified focused on the vasculature and all found disruption of these proteins during diverse disease states, reinforcing the view that a variety of etiologies of critical ill may converge on a common pathway to disrupt the endothelial barrier. This begs more focused and nuanced research.

Critical illness may induce endothelial TJ disruption through mechanical, ischemic, or overwhelming inflammatory insults. Studies have investigated serum levels of claudin-5, ZO-1, occludin, and zonulin in each setting. In mechanical injury, such as polytrauma and trauma-induced hemorrhagic shock, levels of claudin-5 and JAM-A are elevated and correlate with outcomes. The prognostic value of serum claudin-5 in hemorrhage conversion of ischemic stroke highlights its utility as a marker of endothelial barrier breakdown following mechanical and ischemic damage. Inflammatory stimulation may lead to the active disassembly of endothelial TJs, as is presumed to occur in capillary leak associated with septic shock. Serum ZO-1, occludin, and zonulin are all elevated in sepsis; however, ZO-1 best stratified sepsis severity and degree of organ dysfunction. That ZO-1 outperformed other TJ markers may reflect the concomitant organ epithelial injury which occurs in multiple organ dysfunction syndrome. Similarly, in Dengue fever, serum levels correlated with the amount of plasma leakage, demonstrating its specificity as a biomarker of vascular barrier dysfunction.

A number of clinical factors may initiate destabilization of epithelial and endothelial barriers in critical illness, such as overwhelming or unrelenting stimulation from inflammatory (i.e., cytokines), hypoxic-ischemic or traumatic insults (i.e., damage-associated molecular patterns). Yet, regardless of the particular instigating factors, injured endothelial and epithelial TJs may share a common molecular pathway of active disassembly [4, 65]. Despite an increasingly thorough understanding of the molecular mechanisms of active TJ breakdown in cell culture [10], very few therapies developed in vitro have shown a translatable clinical impactful. These deficiencies are most notable in sepsis, a hallmark of which is endothelial dysfunction leading to capillary leak [66]. Many anti-cytokine therapies, such as antibodies that target vascular endothelial growth factor or neutralize lipopolysaccharide signaling, have not shown clinical benefit, likely due to complex, time-dependent, and overlapping pathways. Recent trials involving agents that may tighten vascular barriers, such as those that modulate vasopressin [67, 68] and adrenomedullin pathways [69, 70], are more promising. It is possible that these therapies may counter-act the systemic signaling that leads to TJ disruption in endothelium and epithelium, although additional translational research is required.

To date, there has been minimal investigation on the use of TJ markers in cardiac or pulmonary failure, a glaring lapse given its high prevalence and the associated morbidity and mortality. Given the prevalence, morbidity, and mortality of cardiopulmonary disease, efforts to identify specific tissue markers or more rigorously exploiting those already known could lead to clinically useful tools. The histologic (IHC and EM) and molecular (ELISA, WB, and RT-PCR) tests utilized in these studies are not clinically available; however, they are available in reliable kits or arranged in coordination with pathology departments. Availability of such tools may provide opportunities for clinicians, with ready access to patient samples, to conduct clinical research on the utility of TJ-derived markers in challenging clinical scenarios. Unfortunately, there is no data comparing different analytical methods (e.g., investigating claudin-3 in urine with a WB or ELISA), to guide researchers, nor have many of these tests been validated in the clinical setting. Identifying the optimal detection methods for TJ proteins, and adapting such tests to the appropriate clinical specimens, present opportunities to advance this line of research. Ultimately, markers of TJ disruption may have the potential to provide clinicians with more precise information to assist in the diagnosis, management, and improved pathophysiologic understanding of diseases such as capillary leak, pulmonary edema, and multiple organ dysfunction syndrome.

There are limitations to this investigation. As we have noted, multiple proteins (e.g., ZO-1, zonulin, occludin, and others) are expressed in endothelial, epithelial, as well as other types of cells. Accordingly, the origin of cellular injury must be interpreted within the various clinical scenarios, which presents challenges in assessing individual studies and as well as comparing results between them. Nonetheless, due in part to a paucity of qualifying studies, we combined multiple pathologies across age ranges, from infants with NEC to adults with SAP all while being cognizant of the multiple factors of timing and technique that could affect the results. In spite of the challenges, we felt it was important to try to understand the range of clinical settings in which TJ-derived biomarkers might be informative. Unfortunately, because of the great variability in methods of assessment, disease state, and results reporting, we are unable to conduct a quantitative meta-analysis. In many cases, interpretation of pathology specimens was qualitative and primary data could not be completely reviewed. The timing of histologic sampling was inconsistently related to disease onset. In addition, we did not consider functional MRI studies of blood-brain barrier function, gut functional permeability studies, trans-pulmonary water or glycocalyx marker studies due to their lack of direct assessment of TJ structure and function or TJ-derived proteins. Our review may further be limited by the potential impact of publication bias, as studies not finding a correlation between TJ disruption and critical illness would be less likely to be published. Although we attempted to identify all relevant studies, it is possible that qualifying studies were inadvertently omitted from this systematic review.

## Conclusions

Tight junctions are essential to the integrity of epithelial and endothelial barriers and are often disrupted in critical illness. Accordingly, their protein components may make reliable biomarkers of specific tissue injury. We reviewed 30 heterogeneous studies of TJ structure in critically ill humans which assessed tissue specimens, serum, and urine for levels of TJ-derived proteins. These studies collectively indicated that both epithelial and endothelial barriers are dramatically altered by critical illness. Moreover, initial urine and serum study of tissue-specific proteins have shown potential as powerful diagnostic tools. More investigation, along with standardization of research methods, is required to identify with greater precision the optimal markers of tissue-specific injury and correlate these markers to clinically meaningful outcomes.

## Additional files

Additional file 1: **Table S1.** Protocol for systematic review. This protocol was derived by group consensus prior to initiation of the systematic review. This protocol is registered in the PROSPERO database of systematic reviews (https://www.crd.york.ac.uk/prospero/display_record.php?RecordID=74546). (DOCX 17 kb)

Additional file 2: **Table S2.** Medline (PubMed) search strategy. (DOCX 17 kb)

Additional file 3: **Table S3.** Embase (Ovid) search strategy. (DOCX 20 kb)

## Abbreviations

CKD: Chronic kidney disease
CPB: Cardiopulmonary bypass
CSF: Cerebral spinal fluid
ECC: Extracorporeal circulation
ELISA: Enzyme-linked immunosorbent assay
EM: Electron microscopy
EM: Electron microscopy
GI: Gastrointestinal
HD: Hemodialysis
IF: Immunofluorescence
IHC: Immunohistochemistry
IP: Intestinal permeability
JAM: Junctional adhesion molecule
MODS: Multiple organ dysfunction syndrome
NEC: Necrotizing enterocolitis
NR: Not reported
RT-PCR: Reverse transcription polymerase chain reaction
SAP: Severe acute pancreatitis
TJ: Tight junction
WB: Western blot
ZO: Zonula occludens
