# Supplementary material for: Tight junction structure, function, and assessment in the critically ill: a systematic review
Source: Intensive Care Med Exp. 2018 Sep 26;6:37. doi: 10.1186/s40635-018-0203-4 (PMC6158145; doi:10.1186/s40635-018-0203-4)
Supplement: Supplementary file 1 — Table S1. Protocol for systematic review. This protocol was derived by group consensus prior to initiation of the systematic review. This protocol is registered in the PROSPERO database of systematic reviews (https://www.crd.york.ac.uk/prospero/display_record.php?RecordID=74546). (DOCX 17 kb) [file 40635_2018_203_MOESM1_ESM.docx]

**Additional file 1 Tight junction structure, function and assessment in critically ill humans: Review Protocol**

| Section and topic | Item No | Checklist item |  |  |
| --- | --- | --- | --- | --- |
| ADMINISTRATIVE INFORMATION | | |  |  |
| Title: |  |  |  |  |
| Identification | 1a | **Tight junction structure, function and assessment in critically ill patients** |  |  |
| Update | 1b | This is the initial protocol for this systematic review |  |  |
| Registration | 2 | PROSPERO registration number CRD42017074546 |  |  |
| Authors: |  | David Vermette^1^, Pamela Hu^1^, Melissa Funaro^2^, Janis Glover^2^, Maria Balda, Richard Pierce^1^ |  |  |
| Contact | 3a | 1. Department of Pediatrics, Yale University 2. Yale School of Medicine, Medical Library 3. Kings College London   [David.vermette@yale.edu](mailto:David.vermette@yale.edu), [Pamela.hu@yale.edu](mailto:Pamela.hu@yale.edu), [M.balda@ucl.ac.uk](mailto:M.balda@ucl.ac.uk), [Richard.pierce@yale.edu](mailto:Richard.pierce@yale.edu)  Richard Pierce is the corresponding author:  333 Cedar Street, PO Box 208064  New Haven, CT 06520 |  |  |
| Contributions | 3b | Drs. Vermette and Hu reviewed abstracts and articles for inclusion. Dr. Pierce mediated their review and provided clinical expertise. Dr. Balda provided tight junction expertise. Melissa Funaro and Janis Glover designed and conducted the search and prepared articles for review. All authors contributed to drafting and review of the manuscript. |  |  |
| Amendments | 4 | This protocol represents original work and has no amendments |  |  |
| Support: |  |  |  |  |
| Sources | 5a | Support for this work comes from Yale University Department of Pediatrics departmental funds |  |  |
| Sponsor | 5b | There is no external sponsor or funding support for this work |  |  |
| INTRODUCTION | | |  | Describe the rationale for the review in the context of what is already known |
| Rationale | 6 | This review will consist of two parts. First, we will present an expert review of tight junction structure and function in endothelial and epithelial cells to provide clinicians a pathophysiologic foundation to understand the utility and relevance of tight junction components. Second, we will conduct a systematic review of how tight junction integrity and function are assessed clinically and determine if these assessments correlate to meaningful outcomes. Specifically, we will focus on:  Population: Critically ill humans of all ages.  Intervention: None, we will focus on observational and correlative studies  Comparators: Clinical assessment of tight junction structure and function, either by biologic markers (i.e tight junction proteins in blood, urine or other fluids) or imaging (i.e. tight junction morphology on pathologic specimens). |  |  |
| Objectives | 7 | 1. Review the basic physiology of endothelial and epithelial tight junctions as they relate to critical care. 2. Determine how tight junction structure and function has been assessed in critically ill humans. 3. Determine if the clinical assessments of tight junction structure, function or damage correlates to meaningful outcomes, specifically mortality, ICU length of stay, or days requiring respiratory or cardiac support. |  |  |
| METHODS | | |  | Specify the study characteristics (such as PICO, study design, setting, time frame) and report characteristics (such as years considered, language, publication status) to be used as criteria for eligibility for the review |
| Eligibility criteria | 8 | Eligible studies include those assessing tight junction structure or function in critically ill humans. Articles will be screened for meeting the inclusion criteria; **1) Observational case report, cohort, case control or clinical trials studies 2) Studies involving critically ill human patients and 3) Studies assessing tight junction biomarkers or pathology (i.e. tight junction protein-1, -2 and -3, claudin-1 thought -24, occludin, tricellulin, junctional adhesion molecules-A, -B and -C, zona-occludins-1, zonulin and cingulin).** Studies that will be excluded include those investigating animal or cellular models as well as those that do not assess tight junctional components. |  |  |
| Information sources | 9 | We will use a structured search of the following core biomedical databases; PubMed, Embase, Web of Science and Ovid-Medline. The grey literature sources will be searched using clinical trials databases (ClinicalTrials.gov, WHO Clinical Trials reporter), research grant databases (NIH reporter), patent searches (USPTO search), conference abstracts (Web of Science), thesis (Proquest) and publicly available internet searches (Google Scholar). Update 3/6/2018, after sampling and analysis, the team eliminated Web of Science and Scopus as potential databases to search due to the amount of duplication of search results |  |  |
| Search strategy | 10 | Please see Attachment #1 for our detailed search strategy |  |  |
| Study records: |  |  |  |  |
| Data management | 11a | Data will be managed through the online primary literature screening tool Covidence.org with support from Yale University School of Medicine Library. |  |  |
| Selection process | 11b | Two independent, blinded reviewers will complete a title and abstract screen of the search results. After each reviewer selects abstracts the senior author will mediate any discrepancies. After consensus is reached the two reviewers will independently screen full text articles to see if they meet the inclusion criteria. After each reviewer selects complete articles the senior author will mediate any discrepancies. |  |  |
| Data collection process | 11c | Two independent, blinded reviewers will extract data from each selected study using the data extraction form. After all data is extracted, reviewers will meet with the senior author to synthesize the data into a consensus narrative statement. The quantity and heterogeneity of the data will be assessed. |  |  |
| Data items | 12 | Data will be sought for measures of tight junction proteins **(i.e. tight junction protein-1, -2 and -3, claudin-1 thought -24, occludin, tricellulin, junctional adhesion molecules-A, -B and -C, zona-occludins-1, zonulin and cingulin**) in critically ill humans as biomarkers in bodily fluids **(i.e. blood, serum, plasma, urine, cerebrospinal fluid, stool and bronchiolar lavage)** or on histologic **inspection (i.e. biopsy or post-mortem tissue samples)** |  |  |
| Outcomes and prioritization | 13 | Risk of bias will be assessed by each reviewer during the data extraction process using the Cochrane risk of bias tool included in the data extraction form. Risk of bias will be discussed for each study included at the time of the consensus narrative data synthesis and any discrepancies will be mediated by the senior author. |  |  |
| Risk of bias in individual studies | 14 | Data will be qualitatively synthesized as described above. Statements regarding risk of bias in individual studies will be provided. |  |  |
| Data synthesis | 15a | No quantitative or meta-analyses are planned. |  |  |
| Confidence in cumulative evidence |  | A statement on the on the quality of the cumulative evidence will be provided with qualitative details provided in the final manuscript. |  |  |
