# Supplementary material for: Tight junction structure, function, and assessment in the critically ill: a systematic review
Source: Intensive Care Med Exp. 2018 Sep 26;6:37. doi: 10.1186/s40635-018-0203-4 (PMC6158145; doi:10.1186/s40635-018-0203-4)
Supplement: Supplementary file 2 — Table S2. Medline (PubMed) search strategy. (DOCX 17 kb) [file 40635_2018_203_MOESM2_ESM.docx]

Additional file 2: Table S2 MEDLINE (Ovid) search strategy

| 1 exp Tight Junctions/ |
| --- |
| 2 tight junction*.tw. |
| 3 Zonula Occluden*.tw. |
| 4 Zona Occluden*.tw. |
| 5 Occluding Junction*.tw. |
| 6 exp Tight Junction Proteins/ |
| 7 claudin*.tw. |
| 8 Junctional Adhesion Molecule*.tw. |
| 9 Occludin.tw. |
| 10 Tricellulin.tw. |
| 11 Zonulin.tw. |
| 12 Cingulin.tw. |
| 13 ZONAB.tw. |
| 14 TIAM-1.tw. |
| 15 ZO-1.tw. |
| 16 ZO-2.tw. |
| 17 ZO-3.tw. |
| 18 (MARVEL* adj5 protein*).tw. |
| 19 exp Critical Illness/ |
| 20 (critical illness* or critically ill).tw. |
| 21 exp Thoracic Injuries/ |
| 22 (thoracic Injur* or chest Injur*).tw. |
| 23 exp Myocardial Infarction/ |
| 24 (myocardial infarct* or myocardia infarct* or Heart attack* or cardiovascular failure or Cardiogenic shock).tw. |
| 25 exp Stroke/ |
| 26 (stroke* or Cerebrovascular Accident* or Brain Vascular Accident* or Apoplexy).tw. |
| 27 exp Meningitis/ |
| 28 (meningitis or Meningitides or Pachymeningitis).tw. |
| 29 exp Encephalitis/ |
| 30 (encephalitis or Brain Inflammation* or Rasmussen Syndrome or Rasmussen Encephalitis or Rasmussen's Syndrome).tw. |
| 31 exp Hydrocephalus/ |
| 32 Hydrocephal*.tw. |
| 33 (Brain Disease*OR Brain Disorder* or Central Nervous System Disorders or Encephalopath*).tw. |
| 34 exp Brain edema/ |
| 35 (brain edema or brain oedema or cerebral oedema or cerebral edema).tw. |
| 36 exp kidney failure, chronic/ |
| 37 (kidney injur* or Renal Injur* or Renal Insufficienc* or Kidney Insufficienc* or Kidney Failure* or Renal Failure* or End Stage Kidney Disease or kidney disease* or renal disease*).tw. |
| 38 exp Renal Insufficiency, Chronic/ |
| 39 exp acute kidney injury/ |
| 40 acute kidney.tw. |
| 41 exp Organ Transplantation/ |
| 42 (organ transplant* or Organ Grafting*).tw. |
| 43 exp burn/ |
| 44 (burn or thermal injur*).tw. |
| 45 exp Respiratory Distress Syndrome, Adult/ |
| 46 (respiratory distress syndrome or lung injury or respiratory failure).tw. |
| 47 exp Pulmonary Edema/ |
| 48 (Pulmonary edema* or Pulmonary failure or Wet Lung*).tw. |
| 49 exp pneumonia/ |
| 50 Pneumonia*.tw. |
| 51 exp Respiration, Artificial/ |
| 52 (Mechanical ventilation* or Artificial Respiration*).tw. |
| 53 exp Status Asthmaticus/ |
| 54 (Status Asthmaticus or Asthmatic Cris* or asthma).tw. |
| 55 exp Extracorporeal Membrane Oxygenation/ |
| 56 (Extracorporeal Membrane Oxygenation* or Extracorporeal membranous oxygenation* or Extracorporeal Life Support* or Extracorporeal).tw. |
| 57 exp Brain Injuries, Traumatic/ |
| 58 (Traumatic Brain Injur* or Brain Trauma* or Traumatic Encephalopath* or cerebral dysfunction).tw. |
| 59 exp Status Epilepticus/ |
| 60 (status epilepticus or hypoxic injur* or hypoxic ischemic injur* or brain injur*).tw. |
| 61 exp Pulmonary Embolism/ |
| 62 (Pulmonary Embol* or Pulmonary Thromboembolism*).tw. |
| 63 exp Sepsis/ |
| 64 (sepsis or septic or Pyemia or Pyohemia or Pyaemia or Septicemia* or Blood Poisoning* or Severe infection*).tw. |
| 65 exp systemic inflammatory response syndrome/ |
| 66 (systemic inflammatory response syndrome or SIRS).tw. |
| 67 exp Bacteremia/ |
| 68 bacteremia*.tw. |
| 69 exp Multiple Organ Failure/ |
| 70 (multiple organ failure* or Multiple organ dysfunction).tw. |
| 71 exp Liver Failure, Acute/ |
| 72 (Acute liver failure* or Fulminating Hepatic Failure* or Fulminant Liver Failure* or Acute Hepatic Failure* or Fulminant Hepatic Failure* or Fulminating Liver Failure*).tw. |
| 73 exp Enterocolitis, Necrotizing/ |
| 74 (Necrotizing enterocolitis or Gastrointestinal failure* or Endothelial dysfunction or Epithelial dysfunction or Capillary leak or Vascular dysfunction or Vascular permeability or Epithelial permeability or Endothelial permeability).tw. |
| 75 exp bacterial translocation/ |
| 76 Bacterial translocation.tw. |
| 77 exp Reperfusion Injury/ |
| 78 (Ischemia injur* or Reperfusion Damage* or Reperfusion Injur* or Bone marrow failure or Thrombocytopenia or Severe anemia).tw. |
| 79 exp Advanced Trauma Life Support Care/ |
| 80 exp Wounds/ and Injuries/ |
| 81 exp Cerebrovascular Trauma/ |
| 82 (cerebrovascular trauma* or vascular injur*).tw. |
| 83 exp Trauma, Nervous System/ |
| 84 (Nervous System Trauma* or Nervous System Injur* or Axonotmesis or Craniocervical Injur* or Neurotmesis).tw. |
| 85 exp Craniocerebral Trauma/ |
| 86 (Craniocerebral Trauma* or head injur* or head trauma*).tw. |
| 87 exp Head Injuries, Closed/ |
| 88 exp Head Injuries, Penetrating/ |
| 89 exp Coma, Post-Head Injury/ |
| 90 coma*.tw. |
| 91 exp Spinal Cord Injuries/ |
| 92 (spinal cord injur* or spinal cord trauma*).tw. |
| 93 exp Abdominal Injuries/ |
| 94 Abdominal Injur*.tw. |
| 95 exp Traumatic Diaphragmatic Hernia/ |
| 96 Traumatic Diaphragmatic Hernia*.tw. |
| 97 exp Splenic Rupture/ |
| 98 exp Stomach Rupture/ |
| 99 (stomach rupture* or gastric rupture*).tw. |
| 100 exp Amputation, Traumatic/ |
| 101 Traumatic Amputation*.tw. |
| 102 (Asphyxia or Suffocation or Barotrauma or Barotraumas).tw. |
| 103 exp Blast Injuries/ |
| 104 Blast Injur*.tw. |
| 105 exp Decompression Sickness/ |
| 106 (Decompression Sickness or Caisson Disease or bends or Sunburn).tw. |
| 107 exp Contrecoup Injury/ |
| 108 Contrecoup Injur*.tw. |
| 109 exp Crush Injuries/ |
| 110 Crush Injur*.tw. |
| 111 exp Crush Syndrome/ |
| 112 (Crush Syndrome* or Drowning*).tw. |
| 113 exp Electric Injuries/ |
| 114 (Electric Injur* or electrocution).tw. |
| 115 exp Multiple Trauma/ |
| 116 Multiple Trauma*.tw. |
| 117 exp Fractures, Multiple/ |
| 118 (multiple fracture* or Retropneumoperitoneum or Rupture*).tw. |
| 119 exp Rotator Cuff Injuries/ |
| 120 Rotator Cuff Injur*.tw. |
| 121 exp Shock, Traumatic/ |
| 122 traumatic shock.tw. |
| 123 exp Flail Chest/ |
| 124 Flail Chest.tw. |
| 125 exp Heart Injuries/ |
| 126 Heart Injur*.tw. |
| 127 lung Injur*.tw. |
| 128 exp Rib Fractures/ |
| 129 Rib Fracture*.tw. |
| 130 exp Peripheral Nerve Injuries/ |
| 131 Peripheral Nerve Injur*.tw. |
| 132 exp Vascular System Injuries/ |
| 133 (Vascular System Injur* or Vascular Injur*).tw. |
| 134 exp Wounds, Nonpenetrating/ |
| 135 (Nonpenetrating Wound* or blunt injur* or Contusion*).tw. |
| 136 exp Wounds, Penetrating/ |
| 137 (penetrating wound* or Decapitation).tw. |
| 138 exp Eye Injuries, Penetrating/ |
| 139 eye injur*.tw. |
| 140 exp Wounds, Gunshot/ |
| 141 gunshot wound*.tw. |
| 142 exp Wounds, Stab/ |
| 143 stab wound*.tw. |
| 144 Battered Child Syndrome.tw. |
| 145 exp Corpse Dismemberment/ |
| 146 Corpse Dismemberment.tw. |
| 147 or/1-18 |
| 148 or/19-146 |
| 149 147 and 148 |
| 150 limit 149 to human |
