# Supplementary material for: Tight junction structure, function, and assessment in the critically ill: a systematic review
Source: Intensive Care Med Exp. 2018 Sep 26;6:37. doi: 10.1186/s40635-018-0203-4 (PMC6158145; doi:10.1186/s40635-018-0203-4)
Supplement: Supplementary file 3 — Table S3. Embase (Ovid) search strategy. (DOCX 20 kb) [file 40635_2018_203_MOESM3_ESM.docx]

Additional file 3: Table S3. Embase (Ovid) search strategy

| 1 exp tight junction/ |
| --- |
| 2 tight junction*.tw. |
| 3 Zonula Occluden*.tw. |
| 4 Zona Occluden*.tw. |
| 5 Occluding Junction*.tw. |
| 6 exp tight junction protein/ |
| 7 claudin*.tw. |
| 8 Junctional Adhesion Molecule*.tw. |
| 9 Occludin.tw. |
| 10 Tricellulin.tw. |
| 11 Zonulin.tw. |
| 12 Cingulin.tw. |
| 13 ZONAB.tw. |
| 14 TIAM-1.tw. |
| 15 ZO-1.tw. |
| 16 ZO-2.tw. |
| 17 ZO-3.tw. |
| 18 (MARVEL* adj5 protein*).tw. |
| 19 exp critical illness/ |
| 20 (critical illness* or critically ill).tw. |
| 21 exp thorax injury / |
| 22 (thoracic Injur* or chest Injur*).tw. |
| 23 exp heart infarction/ |
| 24 (myocardial infarct* or myocardia infarct* or Heart attack* or cardiovascular failure or Cardiogenic shock).tw. |
| 25 exp cerebrovascular accident/ |
| 26 (stroke* or Cerebrovascular Accident* or Brain Vascular Accident* or Apoplexy).tw. |
| 27 exp meningitis/ |
| 28 (meningitis or Meningitides or Pachymeningitis).tw. |
| 29 exp encephalitis/ |
| 30 (encephalitis or Brain Inflammation* or Rasmussen Syndrome or Rasmussen Encephalitis or Rasmussen's Syndrome).tw. |
| 31 exp hydrocephalus/ |
| 32 Hydrocephal*.tw. |
| 33 (Brain Disease*OR Brain Disorder* or Central Nervous System Disorders or Encephalopath*).tw. |
| 34 exp brain edema/ |
| 35 (brain edema or brain oedema or cerebral oedema or cerebral edema).tw. |
| 36 exp chronic kidney failure/ |
| 37 (kidney injur* or Renal Injur* or Renal Insufficienc* or Kidney Insufficienc* or Kidney Failure* or Renal Failure* or End Stage Kidney Disease or kidney disease* or renal disease*).tw. |
| 38 exp kidney failure/ |
| 39 exp kidney injury/ |
| 40 acute kidney.tw. |
| 41 exp organ transplantation/ |
| 42 (organ transplant* or Organ Grafting*).tw. |
| 43 exp burn/ |
| 44 (burn or thermal injur*).tw. |
| 45 exp respiratory distress syndrome / |
| 46 (respiratory distress syndrome or lung injury or respiratory failure).tw. |
| 47 exp lung edema/ |
| 48 (Pulmonary edema* or Pulmonary failure or Wet Lung*).tw. |
| 49 exp pneumonia/ |
| 50 Pneumonia*.tw. |
| 51 exp artificial ventilation/ |
| 52 (Mechanical ventilation* or Artificial Respiration*).tw. |
| 53 exp asthmatic state/ |
| 54 (Status Asthmaticus or Asthmatic Cris* or asthma).tw. |
| 55 exp extracorporeal oxygenation/ |
| 56 (Extracorporeal Membrane Oxygenation* or Extracorporeal membranous oxygenation* or Extracorporeal Life Support* or Extracorporeal).tw. |
| 57 exp traumatic brain injury/ |
| 58 (Traumatic Brain Injur* or Brain Trauma* or Traumatic Encephalopath* or cerebral dysfunction).tw. |
| 59 exp epileptic state / |
| 60 (status epilepticus or hypoxic injur* or hypoxic ischemic injur* or brain injur*).tw. |
| 61 exp lung embolism / |
| 62 (Pulmonary Embol* or Pulmonary Thromboembolism*).tw. |
| 63 exp sepsis/ |
| 64 (sepsis or septic or Pyemia or Pyohemia or Pyaemia or Septicemia* or Blood Poisoning* or Severe infection*).tw. |
| 65 exp systemic inflammatory response syndrome/ |
| 66 (systemic inflammatory response syndrome or SIRS).tw. |
| 67 exp bacteremia/ |
| 68 bacteremia*.tw. |
| 69 exp multiple organ failure/ |
| 70 (multiple organ failure* or Multiple organ dysfunction).tw. |
| 71 exp acute liver failure/ |
| 72 (Acute liver failure* or Fulminating Hepatic Failure* or Fulminant Liver Failure* or Acute Hepatic Failure* or Fulminant Hepatic Failure* or Fulminating Liver Failure*).tw. |
| 73 exp necrotizing enterocolitis/ |
| 74 (Necrotizing enterocolitis or Gastrointestinal failure* or Endothelial dysfunction or Epithelial dysfunction or Capillary leak or Vascular dysfunction or Vascular permeability or Epithelial permeability or Endothelial permeability).tw. |
| 75 exp bacterial translocation/ |
| 76 Bacterial translocation.tw. |
| 77 exp reperfusion injury/ |
| 78 (Ischemia injur* or Reperfusion Damage* or Reperfusion Injur* or Bone marrow failure or Thrombocytopenia or Severe anemia).tw. |
| 79 exp advanced trauma life support care / |
| 80 exp wound/ and injury/ |
| 81 exp brain injury/ |
| 82 (cerebrovascular trauma* or vascular injur*).tw. |
| 83 exp nervous system injury/ |
| 84 (Nervous System Trauma* or Nervous System Injur* or Axonotmesis or Craniocervical Injur* or Neurotmesis).tw. |
| 85 exp head injury/ |
| 86 (Craniocerebral Trauma* or head injur* or head trauma*).tw. |
| 87 exp coma/ |
| 88 coma*.tw. |
| 89 exp spinal cord injury/ |
| 90 (spinal cord injur* or spinal cord trauma*).tw. |
| 91 exp abdominal injury/ |
| 92 Abdominal Injur*.tw. |
| 93 exp traumatic diaphragmatic hernia/ |
| 94 Traumatic Diaphragmatic Hernia*.tw. |
| 95 exp spleen rupture/ |
| 96 exp stomach rupture/ |
| 97 (stomach rupture* or gastric rupture*).tw. |
| 98 exp traumatic amputation/ |
| 99 Traumatic Amputation*.tw. |
| 100 (Asphyxia or Suffocation or Barotrauma or Barotraumas).tw. |
| 101 exp blast injury/ |
| 102 Blast Injur*.tw. |
| 103 exp decompression sickness/ |
| 104 (Decompression Sickness or Caisson Disease or bends or Sunburn).tw. |
| 105 exp contrecoup injury/ |
| 106 Contrecoup Injur*.tw. |
| 107 exp crush trauma/ |
| 108 Crush Injur*.tw. |
| 109 exp crush syndrome/ |
| 110 (Crush Syndrome* or Drowning*).tw. |
| 111 exp electric injury/ |
| 112 (Electric Injur* or electrocution).tw. |
| 113 exp multiple trauma/ |
| 114 Multiple Trauma*.tw. |
| 115 exp multiple fracture/ |
| 116 (multiple fracture* or Retropneumoperitoneum or Rupture*).tw. |
| 117 exp rotator cuff injury/ |
| 118 Rotator Cuff Injur*.tw. |
| 119 exp traumatic shock/ |
| 120 traumatic shock.tw. |
| 121 exp flail chest/ |
| 122 Flail Chest.tw. |
| 123 exp heart injury/ |
| 124 Heart Injur*.tw. |
| 125 lung Injur*.tw. |
| 126 exp rib fracture/ |
| 127 Rib Fracture*.tw. |
| 128 exp peripheral nerve injury/ |
| 129 Peripheral Nerve Injur*.tw. |
| 130 exp blood vessel injury/ |
| 131 (Vascular System Injur* or Vascular Injur*).tw. |
| 132 exp blunt trauma/ |
| 133 (Nonpenetrating Wound* or blunt injur* or Contusion*).tw. |
| 134 exp penetrating trauma/ |
| 135 (penetrating wound* or Decapitation).tw. |
| 136 exp eye injury/ |
| 137 eye injur*.tw. |
| 138 exp gunshot injury/ |
| 139 gunshot wound*.tw. |
| 140 exp stab wound/ |
| 141 stab wound*.tw. |
| 142 battered child syndrome.tw. |
| 143 exp corpse dismemberment/ |
| 144 Corpse Dismemberment.tw. |
| 144 or/1-18 |
| 145 or/19-144 |
| 146 147 and 148 |
| 147 limit 149 to human |
